# Quinine Sulphate Microparticles as Treatment for Leishmaniasis

**DOI:** 10.1155/2020/5278518

**Published:** 2020-04-30

**Authors:** Grace Lovia Allotey-Babington, Seth Kwabena Amponsah, Thomas Nettey, Clement Sasu, Henry Nettey

**Affiliations:** ^1^Department of Pharmaceutics and Microbiology, School of Pharmacy, University of Ghana, P.O. Box LG 43 Legon, Accra, Ghana; ^2^Department of Pharmacology and Toxicology, School of Pharmacy, University of Ghana, P.O. Box LG 43 Legon, Accra, Ghana

## Abstract

**Background:**

Leishmaniasis is a neglected tropical disease caused by the *Leishmania* parasite and transmitted by the female phlebotomine sandfly. The disease can affect the skin (least fatal) or internal organs (most fatal). Current treatment options for leishmaniasis have a number of adverse effects, and there appears to be resistance by the protozoan parasite (*Leishmania* spp.). Reports suggest that quinine sulphate, not indicated for leishmaniasis, is effective in killing the *Leishmania* parasite. Indeed, the efficacy of any drug is dependent on the concentration at the target site, which is also almost dependent on drug formulation. The current study assessed the pharmacokinetic profile of the microparticulate formulation of quinine sulphate and its *in vitro* and *in vivo* efficacy against *Leishmania donovani*.

**Methods:**

Quinine sulphate was encapsulated in bovine serum albumin by the spray-drying method. Quinine sulphate microparticles were evaluated for size, zeta potential, drug content, encapsulation efficiency, and *in vitro* release properties. Afterwards, the pharmacokinetic characteristics of quinine sulphate microparticles were estimated and *in vivo* efficacy studies were also conducted.

**Results:**

The size range of the quinine sulphate microparticles was between 2.0 and 5.0 *µ*m. Microparticles had an average zeta potential of −35.2 mV and an encapsulation efficiency of 94.5%. Also, *C*_max_, *t*_1/2_, and AUC were all significantly desirable for quinine sulphate microparticles compared to the drug powder. Quinine sulphate microparticles significantly reduced parasite load in rat organs than amphotericin B.

**Conclusion:**

Overall, quinine sulphate microparticles had better pharmacokinetic profile and showed higher efficacy against *Leishmania donovani* parasites *in vivo*. Thus, quinine sulphate microparticles have the potential, especially, in treating visceral leishmaniasis.

## 1. Introduction

Leishmaniasis, a neglected tropical disease, is known to cause morbidity in more than 1 billion people worldwide [1]. According to the World Health Organization (WHO), leishmaniasis is one of the major causes of death in a number of resource-poor countries [2]. About 94% of new cases of leishmaniasis reported by the WHO in 2017 occurred in Africa, South America, and Asia, making it a disease of global concern [3].

Leishmaniasis is caused by parasites from the genus *Leishmania*, a subclass of the family Trypanosomatidae [4]. At least 20 *Leishmania* species (*Leishmania donovani*, *Leishmania infantum*, etc.) are known to infect humans, and more than 90 sandfly species are known to transmit this parasite after a bite. Transmission of *Leishmania* species can be from humans to animals and/or other humans (anthroponotic) or from animals to humans (zoonotic) [5]. Leishmaniasis presents in 3 major forms: visceral leishmaniasis (highly fatal), also called kala-azar, which can destroy internal organs such as the liver, spleen, and lungs; mucocutaneous leishmaniasis (moderately fatal), which affects mainly mucous membranes; and cutaneous leishmaniasis (least fatal), which mostly affects the skin and could lead to skin sores [6]. For many years, antimicrobial agents, notably the pentavalent antimonials, have been the mainstay treatment options for leishmaniasis [7], however, with time the obligate intracellular parasites became resistant to these agents. Currently, drugs like miltefosine, paromomycin, and amphotericin B are the gold standard in the treatment of leishmaniasis [8, 9]. Paromomycin and amphotericin B are administered parenterally. This mode of administration is invasive, painful, and requires the assistance from trained medical personel; thus, patient compliance is often poor. Miltefosine, an orally approved treatment for leishmaniasis [10], is relatively expensive and has adverse effects on the reproductive and gastrointestinal systems [11]. There have been recent reports that suggest treatment failures with the use of miltefosine and amphotericin B (both deoxycholate and liposomal forms) [9]. Furthermore, resistance to some previously used agents has been reported to be due to long treatment duration and missed drug treatment regimens among patients (probably due to the cost of some of the drugs) [12, 13]. Additionally, a number of the antimicrobials used currently in leishmaniasis treatment have narrow therapeutic indices and thus have a high tendency to cause toxicity [14–16]. There is, therefore, the need to find other agents that are effective against the *Leishmania* parasite and also with high benefit to risk ratio.

In a publication by Nettey et al. in 2016, quinine sulphate, among other antimicrobials, was found to inhibit the growth of *Leishmania* parasites *in vitro*. Quinine sulphate was found to be more effective than two standard antileishmania drugs, pentamidine and amphotericin B *in vitro* [17]. Quinine sulphate, however, has a short half-life and often requires frequent dosing. To recommend quinine sulphate as a drug of choice for the treatment of leishmaniasis, an improved delivery system needs to be developed to reduce the dosing frequency, amongst others, as this disease could be chronic.

Microparticulate drug technology is a science that adapts formulation of drugs into minute particles ranging from 0.1 to 100 *μ*m in size. These drug particles exhibit unique physicochemical properties such as ultrasmall size, large surface to mass ratio, high reactivity, and unique interactions with biological systems. Additionally, these peculiar characteristics are known to improve drug absorption, deliver an adequate amount of drugs to cells of interest, and prolong systemic effects of drugs. Thus, the quest to maximize efficiency and reduce dosing frequency of drugs can be attained with microparticulate formulations [18].

In the current study, we formulated a controlled delivery system (microparticles) of quinine sulphate in an attempt to deliver quinine sulphate to target sites, maintain the desired concentration over time, and improve the efficacy of the drug. To evaluate this, the *in vitro* and *in vivo* antileishmania activities of quinine sulphate microparticles were evaluated.

## 2. Materials and Methods

### 2.1. Test Organism and Reagents

The test organism *Leishmania donovani* (WHO strain DD8) was a gift from Dr. Neelo Singh of the Leishmania Research Society, India. Quinine sulphate standard was a gift from the Centers for Disease Control and Prevention, Atlanta, GA, USA. Bovine serum albumin (BSA) powder, culture media (M199), Alamar blue, glutaraldehyde, sodium bisulfite, and all other reagents used for experiments were purchased from VWR International (Radnor, PA, USA).

### 2.2. Equipment

The Bilon-6000 Y Mini Spray Dryer was purchased from Shanghai Bilon Instrument Co., Ltd. Shanghai, China. The Malvern Zetasizer Nano ZS (for zeta potential analysis) was obtained from Malvern Instruments Inc., Westborough, MA. USA. The Phenom Desktop scanning electron microscope (SEM) was obtained from ThermoFisher Scientific, Phoenix, AZ, USA. Dissolution Apparatus I was obtained from Distek Inc. (North Brunswick, New Jersey, USA). The fluorescence plate reader was obtained from Tecan® Infinite 200 PRO, Mannedorf, Switzerland.

### 2.3. Preparation and Characterization of Quinine Sulphate Microparticles

#### 2.3.1. Preparation of Microparticles

A homogenous mixture of quinine sulphate and bovine serum albumin (BSA) was prepared in the ratio of 1 : 4. Briefly, the BSA solution was first prepared by completely dissolving 4 g of BSA powder in 40 mL of distilled water. Quinine sulphate solution (0.2 g/mL) was slowly added and stirred. Solubility of quinine sulphate was enhanced by the addition of 0.1 N hydrochloric acid dropwise. The final solution was made up to 50 mL with distilled water and allowed to equilibrate at room temperature after constant stirring for two hours. The resulting mixture was then cross-linked with 400 *µ*L of glutaraldehyde solution for an hour at room temperature. Excess glutaraldehyde was neutralized by the addition of 1% sodium bisulphite. The mixture was then spray-dried using a Bilon-6000Y Laboratory Spray Dryer to obtain quinine sulphate loaded microparticles. Microparticle controls (empty microparticles without quinine sulphate) were prepared by spray-drying BSA solution cross linked with glutaraldehyde.

#### 2.3.2. Particle Size and Zeta Potential Determination

Particle size distribution and zeta potential were determined by weighing and suspending 1 mg of the microparticles in 10 mL of distilled water. Part of the suspension (100 *µ*L) was further diluted to 1 mL with distilled water, and the size and charge (runs of three independent experiments) were determined using a Malvern Zetasizer Nano-ZS (Malvern Instruments Inc., USA). The equipment utilizes the principle of light scattering to measure the size of microparticles.

#### 2.3.3. Surface Morphology of Microparticles

To determine surface morphology of the microparticles, a scanning electron microscope (ThermoFisher Scientific, USA) was used. Briefly, microparticles were captured on carbon sheets and observed at 5 kV.

#### 2.3.4. Drug Loading of Microparticles

To determine the amount of quinine sulphate in the microparticles, an estimated 10 mg of the formulation (weighed in triplicate) was crushed in a mortar. Phosphate-buffered saline (PBS, pH 7.4) was added, and the contents were transferred into Eppendorf tubes and centrifuged at 10,000 rpm for 10 minutes. A portion of the supernatant was pipetted and further diluted in PBS to obtain an expected concentration of 20 *µ*g/mL. Quinine sulphate in the microparticles was estimated using a UV spectrophotometer at a wavelength of 334 nm.

#### 2.3.5. Encapsulation Efficiency of Microparticles

The effectiveness of the polymer in entrapping quinine sulphate was determined by calculating the encapsulation efficiency. Encapsulation efficiency is determined as a percent of the theoretical drug content. Mathematically, it is estimated as

(1) $$\begin{array}{c} \%\text{ encapsulation efficiency}=\frac{\text{actual drug loading}}{\text{expected drug loading}}\;\times 100. \end{array}$$

#### 2.3.6. *In Vitro* Drug Release

In determining *in vitro* drug release, 30 mg of the prepared quinine sulphate microparticles was loaded into hard gelatin shells. Equal amounts of quinine sulphate powder were similarly encapsulated and used as control. Six dissolution vessels were filled with 500 mL of phosphate buffer, pH 6.80. Triplicates of the 2 formulations were placed in baskets and allowed to rotate at 100 rpm at 37.1°C, at 0, 30, 60, 120, 240, 480, 720, and 1440 minutes. 5 mL samples were drawn from the outer dissolution flasks and replaced with equal volume of phosphate buffer. The contents of the triplicates of the 2 formulations were assayed using a UV spectrophotometer at a wavelength of 334 nm.

### 2.4. Effect of Quinine Sulphate Microparticles on Intramacrophage *Leishmania donovani* (*In Vitro*)

A sample of *Leishmania donovani* promastigotes was cultured in an M199 culture medium and incubated at 25°C. Growth of parasite was monitored daily using the Neubauer counting chamber to capture the stationary phase of growth. The concentration of the parasites was determined by the formula:

(2) $$\begin{array}{c} N=\left(\text{av}\right)\;\times \;16\;\times \;10^{4}\;\text{cells}/\text{mL}, \end{array}$$

where “*N*” is the concentration of parasites and “av” is the average number of cells.

Growth monitoring was continued until the stationary phase of the parasites was at a concentration of about 4 × 10^7^– 5 × 10^7^ cells/mL.

Rat peritoneal macrophages, which were to be the *in vitro* cells used, were cultured in an M199 culture medium and incubated at 37°C and 5% CO_2_. Growth of macrophages was monitored until they were about 80% confluent. Subsequently, 12-chamber well plates were seeded with 2 × 10^5^ cells/well of macrophages in 1 mL of media and incubated overnight (to aid in equilibration). Macrophages were then infected with *Leishmania donovani* by aspirating the growth media from the wells and overlaying the macrophage cells with 300 *µ*L suspension containing  6 × 10^6^ cells of *L. donovani*. Plates were incubated at 37°C to allow for the parasites to invade the macrophages. After 24 hours, the well plates were washed three times with M199 to remove uninvaded parasites.

The *Leishmania*-infected macrophages were then given various treatments. The first group which served as the negative control was treated with only culture media. The second group was treated with twice the IC_50_ concentrations of amphotericin B solution [17] to serve as positive control. Quinine sulphate solution and quinine sulphate microparticle suspension were prepared in stock concentrations of 10 mg/mL. The stock preparations were further diluted with culture media to obtain a concentration of 6.25 *µ*g/mL, respectively. The third group received quinine sulphate solution (6.25 *µ*g/mL), while the fourth group received quinine sulphate microparticle suspension (6.25 *µ*g/mL). The number of wells per group was 5 (*n* = 5). After 24 hours of incubation (at 37°C), the wells of the plates were washed twice with PBS (pH 7.4). Care was taken not to wash-off the cells at the bottom of the wells. All the groups were then incubated with fresh culture media for another 24 hours. Following that, cells were washed twice with PBS and macrophages fixed with paraformaldehyde for 5 minutes. The wells were then washed again with PBS and PBS with 0.1% Triton X (PBST) for 5 and 15 minutes, respectively, to eliminate the paraformaldehyde. The plates were further incubated in 4′6′-diamidino-2-phenylindole for 10 minutes, after which they were once again washed with PBS and PBST. The parasite count in each well was determined with a fluorescent microscope at a magnification of 40x. Readings were done by 2 microscopists, and the average of readings was recorded.

### 2.5. Pharmacokinetic Profile of Quinine Sulphate Microparticles

#### 2.5.1. Ethical Statement for Animal Use

The protocol for the study was approved by the Centre for Plant Medicine Research (CPMR), Mampong, Ghana (approval number CPM/A.95/SF.6/111). The European Community guidelines, as accepted principles for the use of experimental animals, were adhered to.

#### 2.5.2. Animal Care

Male Sprague-Dawley (SD) rats with body weight ranging between 174 and 211 g and 6-7 weeks old were used. The animals were obtained from the Animal House, Centre for Plant Medicine Research (CPMR), Mampong Akuapem, Eastern Region, Ghana. The rats were kept in an environmentally controlled breeding room (temperature 26 ± 2°C and a 12 h dark/light cycle) for 1 week. This was to make animals acclimatize with the environment before experimentation was started. Animals were fed with standard laboratory chow and given water *ad libitum*. All animal treatments and handling were done in accordance with guidelines published by the National Institute of Health for the Care of Laboratory Animals.

#### 2.5.3. Drug Administration and Blood Sampling

To determine the pharmacokinetic parameters of quinine sulphate microparticles compared to quinine sulphate powder formulation, SD rats were randomly put into two groups of five. Quinine sulphate powder was administered to rats in Group 1, while those in Group 2 were given quinine sulphate microparticle suspension. All injections were administered via the intraperitoneal (IP) route using a 23-gauge needle. Each rat was dosed either with 30 mg/kg of quinine sulphate solution or microparticles. Serial tail vein blood samples were collected predose and then at 0.5, 1.5, 2, 4, 8, 12, 24, 48, and 72 hours following IP administration of a single dose of drug solution or microparticles. Each sample was collected into EDTA tubes and stored at −20°C until analyzed.

#### 2.5.4. Estimation of Quinine Sulphate Level in Blood

Frozen blood samples were thawed, and 100 *µ*L was pipetted into Eppendorf tubes. Distilled water (200 *µ*L) was added to each tube and vortex-mixed. A volume of 400 *µ*L of diethyl ether [19] was then added, mixed, and kept at room temperature for 10 minutes. This was later centrifuged at 10,000 rpm for 5 minutes. The upper (organic) layer was transferred into separate Eppendorf tubes and evaporated at 65°C in a water bath. The dried sample was reconstituted with 300 *µ*L of 0.1 M H_2_SO_4_, and 200 *µ*L was transferred into 96-well plates. Samples were then analyzed under fluorescence at 355 nm excitation and 450 nm emission. Drug standards were prepared by spiking whole blood with various concentrations of quinine sulphate, and extraction was done using the same method as described earlier. Both standards and samples were analyzed on the same plate.

### 2.6. Effect of Quinine Sulphate Microparticles on *Leishmania donovani*-Infected Rats

#### 2.6.1. Animal Care and Drug Preparation

Six-week-old Sprague-Dawley (SD) rats (weighing 190 ± 20 g) were obtained from the Centre for Plant Medicine Research (CPMR), Mampong, Ghana. The animals were kept in a pathogen-free facility and handled according to institutional guidelines, under a 12/12 hour light/dark cycle, at a temperature of 26 ± 2°C. Rats were fed with a standard diet and given water *ad libitum*. Quinine sulphate solution and quinine sulphate microparticle suspension were prepared with normal saline to obtain final concentrations of 1 mg/mL.

#### 2.6.2. Infection of Rat with *L. donovani* and Treatment with Quinine Sulphate Formulations

Sprague-Dawley rats, 25 in number (each weighing 190 ± 20 g), were administered intraperitoneally 0.2 mL suspension of 2 × 10^7^ cells of *Leishmania donovani*. Blood samples were taken weekly to check for serum load of *L. donovani* in the rats. Four weeks after infection, the parasite load (described in detail subsequently) in the blood of rats was determined and recorded (to serve as baseline). After this, the rats were divided randomly into five groups (5 in each group). Each rat in the first group was administered intraperitoneal injections of normal saline. Rats in the second group were administered blank microparticles by the same route. Those in the third group received intraperitoneally 10 mg/kg body weight of quinine sulphate solution. Animals in the fourth group were administered quinine sulphate microparticles corresponding to 10 mg/kg body weight of the entrapped drug. Those in the fifth group received amphotericin B solution at 3 mg/kg body weight (the dose of amphotericin B for treating leishmaniasis), as positive control. For each group, treatment was done every other day for a period of two weeks. On day 14, tail vein blood samples were obtained, and parasite load (described in detail subsequently) was determined. After this, the animals were sacrificed, and their livers and spleens were removed and weighed. Portions of the organs were washed with normal saline, weighed, and homogenized in 10 mL of normal saline using a sterile glass Potter-Elvejhem tissue grinder. The homogenates were centrifuged at 1000 rpm, and the supernatant from these homogenates was analyzed for parasite load (described in detail subsequently).

#### 2.6.3. Parasite Load in Blood and Organs

To determine the concentration of parasites in blood and organs, a standard curve of *L. donovani* concentration was obtained. This was done by first serially diluting a known stock suspension (1 × 10^7^ cells/mL) of *L. donovani* in a 96-well plate with M199. A volume of 10 *µ*L of Alamar blue dye was added to each well and left at 25°C for 4 hours. The ability of viable cells to reduce resazurin (the active component in Alamar blue) to resorufin, a red highly fluorescent compound, was determined by measuring fluorescence at an excitation wavelength of 544 nm and emission wavelength of 590 nm.

To determine the parasite load, the blood cells and tissue cells (samples) had to be lysed to release the intracellular parasites. To do this, equal volumes of each sample (blood or organ suspension (homogenate)) were added separately to the M199 growth medium containing Triton X (0.1%) in different tubes and centrifuged at 3000 rpm to lyse cells of samples. Volumes of 100 *μ*L of the supernatants were transferred into a 96-well plate. A volume of 10 *µ*L of Alamar blue dye was added to each well and left at 25°C for 4 hours. Samples were analyzed under fluorescence using a fluorescence plate reader set at an excitation wavelength of 544 nm and emission wavelength of 590 nm, as done for standard samples. The fluorescence readings were obtained for test samples (after 14 days of treatment). The readings were normalized against readings obtained using samples from non-Leishmania-infected rats. Parasite load of test samples were determined from the standard curve constructed.

## 3. Statistical Analysis

Data were expressed as mean values ± standard error of mean. Dunnett's multiple comparison test was used to compare mean values within various groups at 95% confidence interval (CI). The *t*-test was also used to assess the difference between quinine sulphate microparticles and solution, at certain instances.

The pharmacokinetic parameters of quinine sulphate microparticles and solution in the rat model were derived by assuming that the drug was administered into a single compartment. The maximum quinine sulphate plasma concentration (*C*_max_) and its corresponding time (*T*_max_) were determined by inspection of the concentration-time curves. The elimination rate constant (*K*_el_) was assessed by linear regression analysis of the terminal part of the log plasma concentration-time curve. Area under the drug concentration-time curves (AUC) was calculated by the linear trapezoidal rule. AUC was determined till the last measurement point and was also extrapolated to infinity AUC_0⟶∞_. The elimination half-life (*t*_1/2_) was calculated by equating *t*_1/2*e*_ to 0.693 *K*_el_^−1^. The statistical test of significance was performed on the various pharmacokinetic parameters using an unpaired *t*-test at 95% CI.

## 4. Results

### 4.1. Characterization of Quinine Sulphate Microparticles

#### 4.1.1. Particles Size Distribution, Zeta Potential, and Encapsulation Efficiency

**Figure 1:**
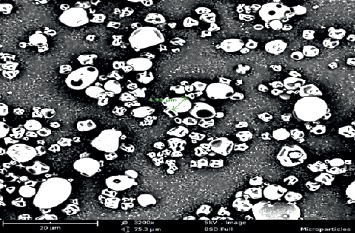
Scanning electron microscope (SEM) images of quinine sulphate microparticles. Scanning electron micrograph of quinine sulphate-BSA microparticles observed at 5 kV. The image shows particles that are irregular and porous.

The average size of quinine sulphate-BSA microparticles obtained ranged between 2.0 and 5.0 *µ*m, with a polydispersity index (PDI) of 0.31. The value of the PDI indicates that the size distribution may be broad. The scanning electron micrograph (Figure 1) showed that the particles were irregularly shaped and porous. The SEM image, additionally, showed a couple of large particles, explaining the value of PDI obtained. The encapsulation efficiency was calculated to be about 95%. Other characteristics of quinine sulphate microparticles are summarized in Table 1.

#### 4.1.2. *In Vitro* Release of Quinine Sulphate Formulations (Microparticles and Powder)

**Figure 2:**
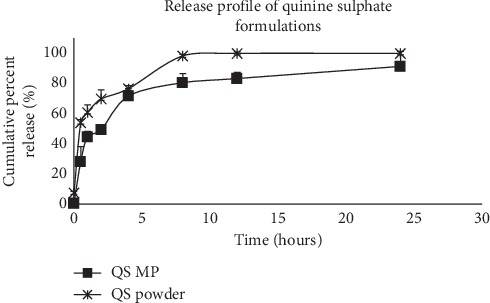
*In vitro* cumulative percent release of quinine sulphate formulations. The microparticle formulation (QS MP) showed extended release profile (91% over 24 hours) compared with the drug powder (QS powder) (98% over 8 hours), *n* = 3. Release of quinine sulphate from the albumin matrix was biphasal. A burst release of about 40% was observed in the first hour.

A high amount (98%) of quinine sulphate was released into the dissolution medium from the powder formulation within the first 8 hours. The release of quinine sulphate from the microparticles was biphasic, with burst release of 40% in the first hour, followed by a continuous steady release of the drug over the next 24 hours. Thus, the matrix used in the formulation of the microparticulate was able to control the release of the drug agent. A cumulative release of 91% of the drug was calculated at the end of the 24 hours (Figure 2). The study was extended to 48 hours, and this yielded an additional release of 8%.

### 4.2. *In Vitro* Activity of Quinine Sulphate Formulations (Microparticles and Powder) against Intramacrophage *L. donovani*

**Figure 3:**
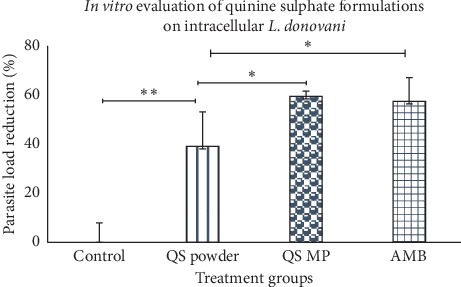
Comparative studies on parasite load reduction in rat peritoneal macrophages using various drug formulations. AMB, amphotericin B; QS powder, quinine sulphate powder; QS MP, quinine sulphate microparticles; Control: untreated group (*n* = 5). Quinine sulphate significantly reduced parasite load in the macrophage cells used. Formulated QS MP were much more effective than the pure quinine sulphate powder. Effect of formulated quinine sulphate microparticles was comparable to the standard drug amphotericin B, the positive control (^*∗*^*p* < 0.05 and ^*∗∗*^*p* < 0.01).

The susceptibility of *L. donovani* amastigotes to quinine sulphate was determined by measuring the percentage reduction of parasites compared to untreated infected control cultures. The results show that all 3 agents used, quinine sulphate powder, quinine sulphate microparticles, and amphotericin B, were able to inhibit the activity of *L. donovani in vitro* (Figure 3). The parasite load reduction in the groups treated with quinine sulphate microparticles and amphotericin B was much higher than that of the quinine sulphate powder group. The difference was found to be statistically significant (*p* < 0.05). Quinine sulphate microparticle formulation was, however, comparable to amphotericin B, the positive control.

### 4.3. Pharmacokinetic Profile of Quinine Sulphate Microparticles and Powder

A calibration curve for quinine sulphate standard had a linearity (*r*^2^) of 0.9932. The concentrations of the quinine sulphate in the individual blood samples were obtained by interpolating from the standard curve.

**Figure 4:**
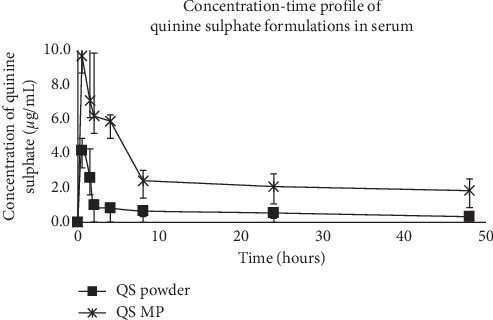
The mean plasma drug concentration-time profile of the quinine sulphate formulations. Sharp spike in serum concentration was observed with the microparticulate formulation (QS MP). Area under the curve (AUC) was significantly higher in the microparticulate formulation group than in the powder group (*p* ≪ 0.01 in Table 2).

The concentration-time curve for quinine sulphate microparticles showed a sharp spike within the first hour after administration compared to the powder (Figure 4). The pharmacokinetic parameters calculated for the two groups are shown in Table 2. All the parameters investigated except *t*_1/2_ were found to differ significantly among the two groups. Peak quinine sulphate blood concentration (*C*_max_) was about 2-fold higher with quinine sulphate microparticles than powder form. Total drug exposure (AUC) was about 3-fold greater with quinine sulphate microparticles than powder form.

### 4.4. Activity of Quinine Sulphate Formulations in *Leishmania donovani*-Infected Rats

**Figure 5:**
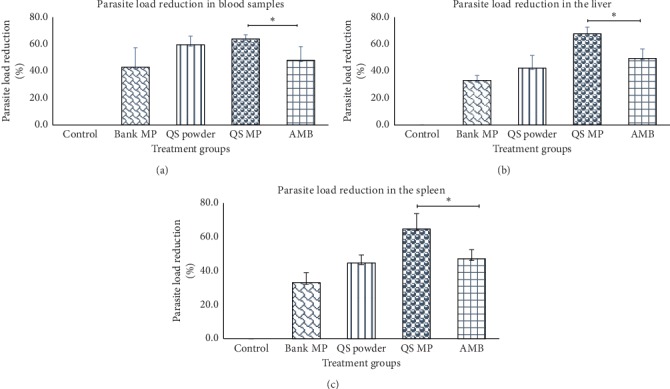
Comparative studies on percent parasite reduction in blood and tissues. In all the tissues studied, treatment with quinine sulphate microparticles resulted in more than 60% reduction in parasite load (*n* = 5). The reduction observed was about 15–18 percentage points better than the standard drug “amphotericin B.” Difference observed was significant (^*∗*^*p* < 0.05). AMB, amphotericin B; QS MP, quinine sulphate microparticles; QS powder, quinine sulphate powder; Blank MP, blank microparticles; Control, untreated group. (a) Blood. (b) Liver. (c) Spleen.

After two weeks of treatment with the various drug formulations, blood samples and tissue samples showed that quinine sulphate microparticle formulation was best at reducing parasite load. Quinine sulphate microparticles were found to significantly (*p* < 0.05) reduce *L. donovani* in blood and tissues (the liver and spleen) of infected rats compared to amphotericin B. The rats treated with quinine sulphate powder eliminated more parasites in the blood than in the tissues. This probably was due to rapid absorption of the unformulated drug (quinine sulphate powder) into circulation. A summary of parasite clearance in blood and tissues (the spleen and liver) with different treatments is shown in Figures 5(a)–5(c).

## 5. Discussion

Quinine sulphate had earlier been shown to be effective, *in vitro*, against *L. donovani* [17]; however, due to its short half-life, frequent dosing will be necessary in order to maintain steady concentrations of the drug in the blood. To reduce this pharmacokinetic challenge, a microparticulate delivery system of quinine sulphate was formulated. Quinine sulphate was entrapped in a BSA matrix that controlled the release of the drug. The average size of particles prepared ranged between 2 and 5 *μ*m, and had a negative zeta potential (−35.2 mV). SEM monograph revealed irregular and porous particles. Particles with such properties usually exhibit good flow in the tablet manufacturing process. The amount of quinine sulphate loaded into the microparticles yielded an encapsulation efficiency of approximately 95%, an indication of the high efficiency of the spray-drying technique.

The release of quinine sulphate from the microparticle polymer matrix occurred in two phases: first, an initial burst release of the unentrapped drug (quinine sulphate that adhered to the outer surface of the matrix during the manufacturing process), followed by a slower continuous release from the polymer matrix due to its hydration and diffusion of drug through pores. The porous nature of the microparticles as confirmed in the scanning electron micrograph image must have contributed significantly to the effective release of the drug (91%) in 24 hours. Kesse et al. conducted a similar study, where they formulated amodiaquine and quinine sulphate microparticles using BSA as their matrix. The average particle size, zeta potential, and *in vitro* release profile they reported were similar to those obtained in this current study [20]. Although the formulation and evaluation of the microparticles prepared are similar to the work done by Kesse et al., this study further investigated the *in vivo* release of the formulated microparticles and conducted efficacy (*in vitro* and *in vivo*) studies against the leishmania parasites.

The pharmacokinetic characteristics of the quinine sulphate formulations were evaluated in SD rats. Formulating the drug into microparticles enhanced the extent of absorption from the site of administration into systemic circulation. This was exhibited by both high *C*_max_ and AUC values for the microparticle formulation compared to those for quinine sulphate powder. The high *C*_max_ of the microparticle formulation, which was achieved in a short time, coupled with the high total drug exposure (AUC) is indicative that the microparticles were absorbed much and to a greater extent from the peritoneal cavity. The microencapsulation matrix used (BSA) affected the way the drug was released into circulation. The degradation process can be categorized as either bulk-eroding or surface-eroding [21–23]. Bulk-eroding polymers exhibit a peculiar phenomenon known as the “burst release” kinetics. Bovine serum albumin (BSA) is a bulk-eroding polymer, explaining the initial rise in the plasma concentration of the drug within the first 30 minutes of administration. Furthermore, the surface drug, which caused 40% burst release in the *in vitro* study, would make quinine sulphate readily available. It may, however, be desirable when high levels of the drug are required to kill parasites, followed by sustained therapeutic levels. Also, the longer elimination half-life (*t*_1/2_) of the microencapsulated quinine sulphate would translate into less frequent dosing.

The percent parasite load in the blood and organs after quinine sulphate administration was determined by finding the percentage of parasite load of the treated cells to that of the untreated cells (negative control). The percent parasite reduction was then determined relative to untreated [24]. Results from this study indicated that quinine sulphate microparticles reduced tissue *L. donovani* levels significantly compared to the positive control (amphotericin B). This outcome confirms findings of previous work by Nettey et al., who demonstrated that amodiaquine (drug) in particulate form had a high potential for the treatment of visceral leishmaniasis [25]. Furthermore, it was observed that blank microparticles, which did not contain any drug, showed some levels of antileishmania activity in the organs compared to the negative control (normal saline). Microparticles by their unique morphology resemble some types to microorganisms, and thus, their presence in the body can stimulate an innate immune response. Phagocytic cells, such as macrophages are attracted to the site to eliminate the foreign substance (microparticles). This immunological response generated could explain the clearance of the parasites by the blank (empty) microparticles.

Quinine sulphate solution was observed to reduce the parasite load in the blood by 42.9%; however, in the tissues, the reduction in parasite load was about 33%. This could be due to the fact that quinine sulphate solution is readily absorbed into circulation. Concentration of the drug solution in the tissues may not have been as high as in the blood to reduce the parasite load. In the groups treated with the microparticulate formulation, a higher reduction of parasites in the blood and tissues were observed. The most plausible explanation for better parasite clearance observed for quinine sulphate microparticles both *in vitro* and *in vivo* may be due to the fact that more quinine sulphate particles may have been engulfed by peritoneal macrophages (phagocytic cells) just after the IP administration. Phagocytic cells usually migrate to the secondary lymphoid organs such as the spleen after taking up foreign substances from the body for further processing before finally ending up in the liver. Due to the fact that these microparticles are loaded with quinine sulphate, the concentration of the drug in these organs tends to be high. This could explain the high reduction in parasite load in the above mentioned organs as compared to the blood. Coincidentally, macrophages are among the cells which serve as hosts to *Leishmania* parasites in the body. A statistically significant difference (*p* < 0.05) in parasite load was observed between the formulated microparticles and the positive control, amphotericin B, after 2 weeks of treatment in both blood and tissues investigated. This can be speculated to be main because of poor absorption of amphotericin B into cells or effective efflux by the cells. Clearing of parasite load in the blood as well as tissue is very critical in eliminating *Leishmania* parasites. This is because parasites left in tissues after treatment could lead to future reinfection.

## 6. Conclusion

Microencapsulation of quinine sulphate using the spray-drying process produced microparticles with an efficient release profile. These properties of the microparticles were exhibited both *in vitro* and *in vivo* (pharmacokinetic and efficacy studies). Additionally, the microparticulate form of quinine sulphate was more effective in clearing intratissue *L. donovani* than the conventional drug (powder form). From the current study, quinine sulphate microparticles showed potential as a drug of choice for leishmaniasis (especially visceral). Quinine sulphate should be further evaluated in human studies as a potential treatment for leishmaniasis. Since quinine sulphate has high oral bioavailability, oral administration of the microencapsulated drug should be further investigated.

## Figures and Tables

**Table 1:** Characteristics of quinine sulphate microparticles.

Average particle size	2–5 *µ*m
Zeta potential	−35.2 mV
Drug loading	18.9%
Entrapment efficiency	94.5%

Average size of microparticles was determined to be between 2 and 5 microns with a polydispersity index (PDI) of 0.31.

**Table 2:** Pharmacokinetic parameters of quinine sulphate formulations.

Pharmacokinetic parameter	QnSO_4_ powder, mean ± SE of mean	QnSO_4_ microspheres, mean ± SE of mean	*p* value	Significantly different (*p* < 0.05)
*C*_max_ (*µ*g/mL)	3.73 ± 0.403	6.81 ± 0.977	0.0194	Yes
*T*_max_ (hr)	0.700 ± 0.200	2.40 ± 0.696	0.0470	Yes
*t*_1/2_ (hr)	9.7 ± 2.4	45.4 ± 16.1	0.0931	No
AUC_0⟶72_ (*µ*g·hr/mL)	50.2 ± 5.40	154 ± 14.6	0.0002	Yes
AUC_0⟶∞_ (*µ*g·hr/mL)	90.9 ± 39.8	266 ± 61.8	0.0443	Yes

Pharmacokinetic properties were higher in the microparticulate formulation of quinine sulphate than in the powder (*n* = 5).

## Data Availability

The data that support the findings of this study are available from the corresponding author upon reasonable request.

## Abbreviations

AUC: Area under the curve
WHO: World Health Organization
GA: Georgia
BSA: Bovine serum albumin
CDC: Centers for Disease Control and Prevention
SEM: Scanning electron microscope
RPM: Rotations per minute
PBS: Phosphate-buffered saline
PBST: Phosphate-buffered saline with Triton X
UV: Ultra violet
CPMR: Centre for Plant Medicine Research
SD: Sprague-Dawley
IP: Intraperitoneal
EDTA: Ethylenediaminetetraacetic acid
CI: Confident interval
*C*_max_: Maximum serum concentration
*T*_max_: Time taken to reach the maximum serum concentration
ANOVA: Analysis of variance
PDI: Polydispersity index
*t*_1/2_: Half-life
*K*_el_: Elimination rate constant
GLAB: Grace Lovia Allotey-Babington
SKA: Seth Kwabena Amponsah
TN: Thomas Nettey
CS: Clement Sasu
HN: Henry Nettey.
